# The genetic regulation of size variation in the transcriptome of the cerebrum in the chicken and its role in domestication and brain size evolution

**DOI:** 10.1186/s12864-020-06908-0

**Published:** 2020-07-29

**Authors:** Andrey Höglund, Katharina Strempfl, Jesper Fogelholm, Dominic Wright, Rie Henriksen

**Affiliations:** 1 AVIAN Behavioural Genomics and Physiology group, IFM Biology, Linköping University, 58183 Linköping, Sweden; 2 Institute of Molecular Regenerative Medicine, Spinal Cord Injury and Tissue Regeneration Center Salzburg (SCI-TReCS), Paracelsus Medical University, Salzburg, Austria; 3 Spinal Cord Injury and Tissue Regeneration Center Salzburg (SCI-TReCS), Paracelsus Medical University, Salzburg, Austria

**Keywords:** eQTL, Cerebrum, Avian, Brain evolution, Hypothalamus, Liver, Domestication

## Abstract

**Background:**

Large difference in cerebrum size exist between avian species and populations of the same species and is believed to reflect differences in processing power, i.e. in the speed and efficiency of processing information in this brain region. During domestication chickens developed a larger cerebrum compared to their wild progenitor, the Red jungle fowl. The underlying mechanisms that control cerebrum size and the extent to which genetic regulation is similar across brain regions is not well understood. In this study, we combine measurement of cerebrum size with genome-wide genetical genomics analysis to identify the genetic architecture of the cerebrum, as well as compare the regulation of gene expression in this brain region with gene expression in other regions of the brain (the hypothalamus) and somatic tissue (liver).

**Results:**

We identify one candidate gene that putatively regulates cerebrum size (*MTF2*) as well as a large number of eQTL that regulate the transcriptome in cerebrum tissue, with the majority of these eQTL being trans-acting. The overall regulation of gene expression variation in the cerebrum was markedly different to the hypothalamus, with relatively few eQTL in common. In comparison, the cerebrum tissue shared more eQTL with a distant tissue (liver) than with a neighboring tissue (hypothalamus).

**Conclusion:**

The candidate gene for cerebrum size (*MTF2*) has previously been linked to brain development making it a good candidate for further investigation as a regulator of inter-population variation in cerebrum size. The lack of shared eQTL between the two brain regions implies that genetic regulation of gene expression appears to be relatively independent between the two brain regions and suggest that coevolution between these two brain regions might be more functionally driven than developmental. These findings have relevance for current brain size evolution theories.

## Background

The cerebrum plays a pivotal role in voluntary behavior and cognition [1, 2]. Birds, as well as mammals, have a much larger cerebrum compared to reptiles and other vertebrates of similar size, indicating that selection for increased size of this brain region has played an important role during avian brain evolution [3]. The avian cerebrum varies in size between species and populations both in terms of absolute size and proportional size within the brain [4–6]. These size differences are believed to reflect differences in functionality with increased size providing more processing power [7, 8].

The underlying mechanisms that control cerebrum size and the extent to which the cerebrum can expand in size irrespectively of expansion of other brain regions and the brain itself, is not well understood. Comparative studies have identified consistent patterns of covariance between brain regions indicating the presence of significant constraint on independent brain regions size evolution [9], and two main theories have attempted to explain these evolutionary patterns; (1) the developmental constraints hypothesis (concerted brain evolution hypothesis) suggests that individual brain regions tend to evolve together because they are limited by the same underlying developmental and genetic mechanisms during neurogenesis, while (2) the functional constraints hypothesis (mosaic brain evolution hypothesis) suggests more complex underlying mechanisms that allow independent development and growth of discrete brains regions and where correlated evolution of brain regions reflects functional constrains. These hypotheses are not mutually exclusive, and previous correlative studies suggest a combination of both mosaic and concerted evolution [10–12], but they make different predictions about brain evolution [9].

We still know very little about the underlying genetic mechanism that control cerebrum size, and in particular about how quantitative variation in cerebrum size is regulated.

To date studies have mainly focused on the genetic programs involved in determining the basic architecture of the cerebrum [13], but the identification of genes regulating differences in cerebrum size and how they differ from gene expression in other brains regions could provide a more direct assessment of the underlying causes of covariance and coevolution between brain regions. During domestication chickens (*Gallus gallus*) developed a significantly larger cerebrum (both absolute and proportional to the rest of the brain) compared to their wild progenitor, the Red junglefowl. Overall brain size also increased during domestication in chickens, with the loci responsible for increasing brain size being distinct from those increasing body size during domestication [5, 14]. We have previously shown that the genomic regions underlying cerebrum size in chickens are separate to the genetic regions underlying other major brain regions in the chicken brain [5] suggesting less common constrain on size evolution between brain regions. The fast alteration to brain size and composition occurring during the reversion from a domestic to a wild state (feralization) also support the notion that alterations in the size of individual brain regions is less constrained by other brain regions than previously thought [15, 16].

In this study, we combine measurement of total as well as proportional (to the rest of the brain) cerebrum with genome-wide genomics analysis to identify the genetic architecture of the transcriptome in the cerebrum using an advanced intercross based on Red junglefowl and domestic White leghorn chickens. These birds have been previously demonstrated to be an excellent model for the genetic regulation of brain size variation due to the large differences in brain size and composition generated via domestication [5]. By combining these results with previous analyses of the genetic regulation of the hypothalamus [17] and liver transcriptomes [18] in the same chicken intercross, we can compare the regulation of gene expression in both brain regions, as well as between the brain and distant somatic tissue. This allows a comparison of the transcriptomic regulation of these different tissues and the relative similarity between them, that can help shed light on the evolutionary pattern of brain evolution. The use of expression quantitative trait locus (eQTL) rather than simply gene expression, is important as we are now identifying actual loci that vary between the two genotypes (i.e. the loci responsible for between-population variation [19]), rather than simply high or low overall gene expression. By combining previously identified phenotypic quantitative trait locus (QTL) for brain size and composition with gene expression QTL (eQTL) analyses [5], we can attempt to identify putative genes underlying the phenotypic differences in overall cerebrum size that separate wild and domestic chickens.

## Results

### The genetic regulation of the cerebrum transcriptome - eQTL mapping

**Fig. 1:**
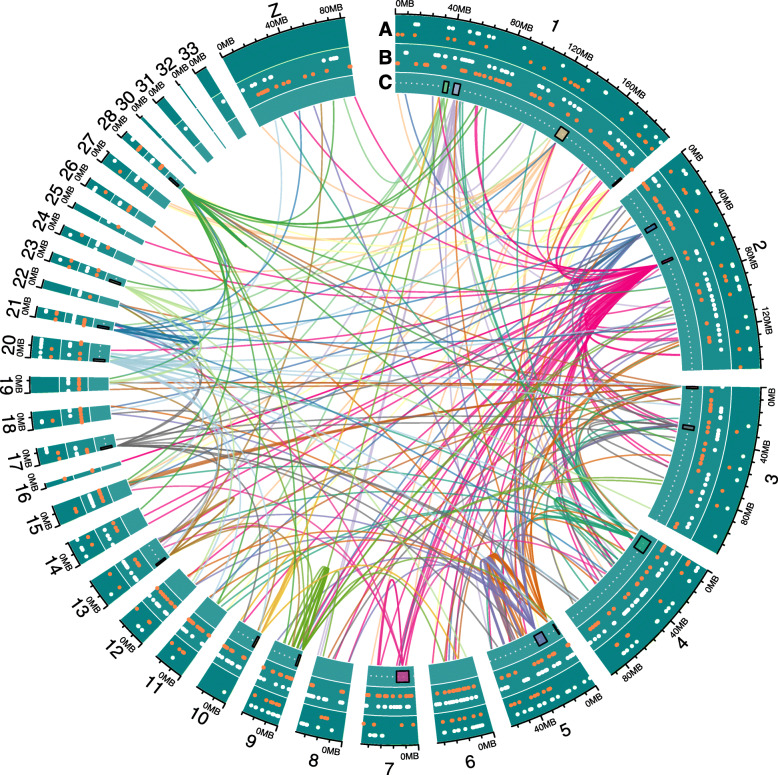
Circular overview of the chicken genome. Track A and B shows cis and trans eQTL respectively. Where each point corresponds to a suggestive eQTL with the QTL effect indicated by white for White Leghorn and red for Red junglefowl allele. Track C shows eQTL hotspot confidence interval with the fan representing the gene location associated with the eQTL

eQTL mapping of cerebrum identified a total of 1315 eQTL. The majority of eQTL were trans acting (1011 trans and 304 cis). An equal distribution of QTL effect was observed, around half of the eQTL had a greater increase in gene expression from the Red junglefowl allele (613) and half had a greater effect from the White Leghorn allele (702), see Table 1 for a summary. Figure 1 shows the eQTL positions and the QTL effects. A total of 840 eQTL had a probeset with an annotated gene, the remaining were expressed sequence tags (EST). For the full list of eQTL see supplementary Table S1.

**Table 1:** eQTL mapping summary

Tissue	eQTL	Type (cis | trans)	QTL direction (RJF | WL)	Sex interaction (sex | non-sex)	Probesets	Annotated genes
Cerebrum	1315	304 | 1011	613 | 702	437 | 878	1286	840
Hypothalamus	1123	1064 | 59	520 | 603	635 | 488	1111	645

### Cerebrum trans eQTL hotspots

A total of 20 eQTL hotspots were identified located on 14 of the autosomal chromosomes, see supplementary Table S2. These eQTL hotspots encompassed 319 genes with the largest hotspot located on chromosome 2 with intervals between 1040 and 1067 cM interacting with 54 genes dispersed over 18 chromosomes and with the majority of QTL effects increasing in the Red Junglefowl genotype (45 RJF and 9 WL QTL), see supplementary Table S2. Furthermore, every eQTL and the corresponding genes present in a hotspot was checked for enrichment using the DAVID database. These enrichments showed that these hotspots were enriched for serine/threonine protein kinase activity (chromosome 1 - with such serine-threonine kinases known to affect behavior), neurogenesis (chromosome 3), and g-protein signaling (chromosome 3), epidermal growth factor signaling (chromosome 4), RNA polyadenylation (chromosome 5), amongst a number of other GO terms (see supplementary Table S2).

### Candidate genes for the regulation of cerebrum size

From a previous study using the advanced intercross, we identified QTL for cerebrum size (absolute and proportional), as well as for body mass, brain mass and for the mass of all other major brain-regions (Optic tectum, Cerebellum and Midbrain [5]). To attempt to identify candidate genes underlying cerebrum size and potential link to the size of other brain regions, brain size or overall body size, we first assessed the number of cerebrum eQTL that overlapped with these phenotypic QTL, with a total of 175 eQTL being present (112 between brain mass and 63 between body mass), see supplementary Table S3. Fifteen of the overlaps were significant on a 5% nominal level (2 for cerebrum size, 1 for optic tectum size and 12 for body mass) and therefore considered suggestive, whilst 5 were significant after undergoing multiple testing and therefore considered significant (all for body mass), see supplementary Table S4. The three suggestive overlaps found for brain-mass-phenotypes were for total and relative size of the cerebrum, and the relative size of the optic tectum. To further test putative causality, the suggestive and significant overlaps were tested with the Network Edge Orientation (NEO) software that assesses causality based on correlations between the traits and the SNP genotypes that anchor them (see methods). Some phenotypic QTL had multiple loci that were associated with the trait, epistatic interactions, and thus all these loci were tested for causality. Three overlaps survived the NEO analysis and were significant; for cerebrum mass the gene *MTF2* (on chromosome 8, leo.nb = 0.33) and for adult body mass the genes *PCBD2* (on chromosome 13, leo.nb = 0.42) and an EST (*603848039F1* on chromosome 4, leo.nb = 0.76), see Table 2.

**Table 2:** Cerebrum eQTL CI overlapping with phenotypic QTL CI

Gene Name	Gene location	Trait	*P*-value	NEO					
				Threshold	Edge	leo.nb.oca	leo.nb.cpa	Model	Overlap
PCBD2	chr13:16352132–16368162	Body mass	0.005	Distal	probeset - > trait	0.421	0.947	0.138	chr4@269–286 | chr6@195–214
*603848039F1*	EST	Body mass	0.04	Distal	probeset - > trait	0.756	0.706	0.0254	chr4@265–332 | chr24@6–18
MTF2 ^a^	chr8:12939656–12962759	Absolute cerebrum size	0.018	Local	probeset - > trait	0.331	0.366	0.00000278	chr8@79–123 | chr8@42–89
*603231821F1*	EST	Body mass	0.001***	NS	probeset - > trait	0.216	0.593	1.18E-09	chr12@43–65 | chr12@45–79
*603231821F1*	EST	Body mass	0.004***	NS	probeset - > trait	−1.84	0.149	1.32E-09	chr4@270–285 | chr4@254–274
GIP	chr27:6090482–6098133	Body mass	0.005***	NS	probeset - > trait	−1.62	−1.62	0.0175	chr27@60–86 | chr27@56–80
ENSGALG00000034065	chr4:83916252–83928157	Body mass	0.005***	NS	probeset - > trait	0.00701	0.0012	8.4E-42	chr1@480–534 | chr1@507–516
SCFD2	chr4:65762016–65941747	Body mass	0.011***	NS	trait - > probeset	−3.27	−0.0137	1.09E-12	chr4@250–290 | chr4@254–274
EPAS1	chr3:26671415–26746226	Relative optic tectum size	0.014	NS	trait - > probeset	−2.86	−0.0183	0.0000187	chr4@219–252 | chr4@201–223
NDUFAF2	chrZ:18970571–19017369	Body mass	0.015	NS	probeset - > trait	−2.37	0.287	1.44E-22	chr4@245–274 | chr4@254–274
PNPLA8	chr1:28651041–28685420	Relative cerebrum size	0.017	NS	probeset - > trait	0.673	0.11	1.04E-28	chr21@4–35 | chr21@0–19
FAM161B	chr5:37899536–37906784	Body mass	0.022	NS	probeset - > trait	−0.7	−0.7	0.0899	chr4@251–274 | chr4@254–274
HDAC11	chr12:6100521–6125075	Body mass	0.025	NS	probeset - > trait	0.368	0.269	8.15E-09	chr12@24–72 | chr12@45–79
HMCES	chr12:9520459–9523459	Body mass	0.037	NS	probeset - > trait	0.01	0.16	0.371	chr12@77–181 | chr12@45–79
C12orf43	chr15:9385682–9389695	Body mass	0.04	NS	probeset - > trait	−1.03	0.107	1.15E-10	chr4@265–284 | chr4@254–274

^a^ Gene retired in galgal6. gene name and location found in galgal4 *** *P*-values significant after multiple testing correction. In the neo-threshold: *distal* - the loci of the phenotypic QTL are all tested against the single loci of the eQTL when at least one of the phenotypic QTL loci is overlapping with the eQTL

### Genetic overlap between cerebrum, hypothalamus and liver tissue

**Fig. 2:**
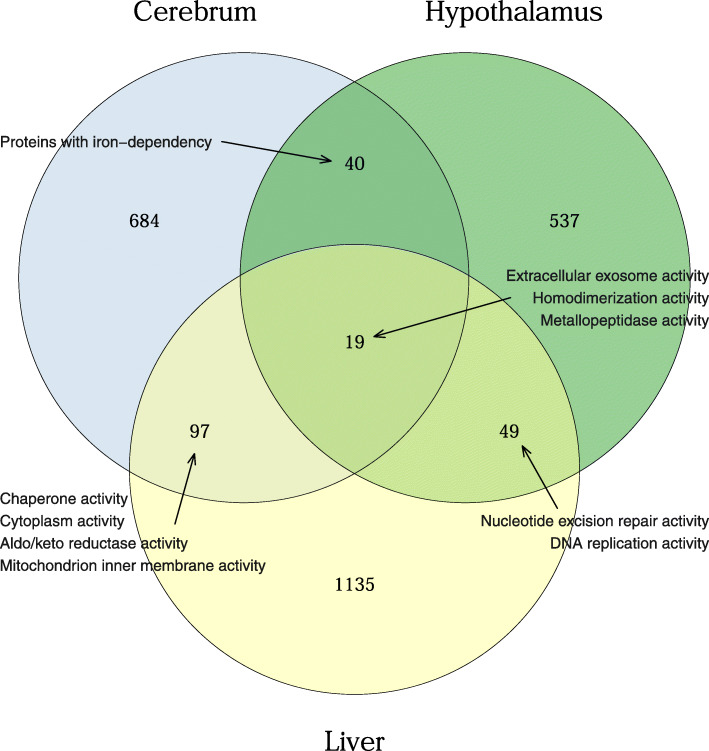
Venn diagram of genes with eQTL found in cerebrum, hypothalamus and liver. Also contains gene ontology terms found for the overlapping genes between the tissues

Previous studies on this advanced intercross have identified eQTL present in hypothalamus and liver. Therefore, to compare the relationship between the gene expression profiles in these different tissues the eQTL identified in each tissue type were analysed. Out of a total of 18,499 probesets, shared between all arrays, 3035 probesets had an eQTL in either of the tissues and 284 probesets had an eQTL in at least two of the tissues. Between cerebrum and hypothalamus 40 genes had an eQTL and between cerebrum and liver 97 genes had an eQTL. Between liver and hypothalamus, 49 genes had an eQTL and between all three tissues 19 genes had an eQTL, see Fig. 2. Therefore, both brain regions shared more eQTL in common with liver tissue than with one another. A GO enrichment analysis was performed on the different eQTL genes present in each of the comparisons (see supplementary Tables S5 and S6). None of these passed an experiment-wide significance threshold (Benjamini-corrected *p*-value < 0.05, corrected for multiple testing), however several passed a suggestive threshold (nominal *p*-value < 0.05, uncorrected for multiple testing). The eQTL genes present in both hypothalamus and cerebrum tissue were suggestively enriched for iron-dependent proteins (nominal *p*-value < 0.027, Benjamini-corrected *p*-value = 0.75), while the eQTL genes shared between hypothalamus and liver tissue were enriched for extracellular exosome activity (*p*-value < 0.029, Benjamini-corrected *p*-value = 0.76), protein homodimerization activity (*p*-value < 0.033, Benjamini-corrected *p*-value = 0.84) and metallopeptidase activity (*p*-value < 0.035, Benjamini-corrected *p*-value = 0.62). The eQTL genes shared between liver and cerebrum tissue were enriched for chaperone activity (*p*-value < 0.028, Benjamini-corrected *p*-value = 0.83), cytoplasm activity (*p*-value < 0.034, Benjamini-corrected *p*-value = 0.95), aldo/keto reductase activity (*p*-value < 0.038, Benjamini-corrected *p*-value = 0.99) and mitochondrion inner membrane activity (*p*-value < 0.039, Benjamini-corrected *p*-value = 0.72). Finally, the eQTL genes shared between all three tissue types were suggestively enriched for P-loop containing nucleoside triphiosphate hydrolase activity (nominal *p*-value < 0.017, Benjamini-corrected *p*-value = 0.38).

### Overlap between eQTL hotspots between cerebrum and hypothalamus tissue

As well as assessing the overlap of eQTL between hypothalamus and cerebrum, the potential overlap between eQTL hotspots present in both tissues was investigated. In total four eQTL hotspot regions overlapped, on chromosome 1 with intervals between 775 and 784 cM, on chromosome 5 with intervals between 243 and 245 cM, on chromosome 10 with intervals between 91 and 97 cM and on chromosome 28 with intervals between 18 and 19 cM. Of these, only the hotspot on chromosome 5 had two genes that shared eQTL in both tissues, *GEMIN2* and *C5H15orf41*. *C5H15orf41* had similar allelic effect in both tissues (i.e. the Red junglefowl allele had the greatest effect in both tissues). For *GEMIN2* the allelic effect was stronger for the White Leghorn allele in cerebrum tissue, while the Red junglefowl allele had the stronger effect in hypothalamus tissue.

## Discussion

In this study we have identified a large number of eQTL that regulate the transcriptome in cerebrum tissue, with the majority of these eQTL being trans-acting. The overall regulation of gene expression variation in the cerebrum was markedly different to the hypothalamus, with relatively few eQTL in common between these two brain regions. In comparison, we find that cerebrum tissue shared more eQTL with a distant tissue (liver) than with the hypothalamus. Furthermore, we also identify one candidate gene that putatively regulates cerebrum size (*MTF2*) and two that potentially regulate overall body mass.

To date, relatively few genes responsible for brain size variation have been identified (chiefly in humans [20–22], mice [23] and chickens [5]). Research into the genes underlying variation in human brain size has identified two loci [24], whilst a large-scale GWAS for intra-cranial volume (maximum brain size) discovered a further five loci [25]. These five loci were identified using extremely large sample sizes (32,438 individuals) to identify association signals in several genes, with these genes involved in neural stem cell proliferation (*FOXO3*) and neurodegeneration (*MAPT*) amongst others. The larger sample size here is somewhat offset by the relatively small variation present in humans compared to the extreme variation generated by comparing wild and domestic birds [5]. Here we have combined this genetics approach, using the loci detected previously, with transcriptomic eQTL to combine actual gene expression with brain size parameters to detect a single candidate for cerebrum size. This is a large advantage over studies that solely rely on association with no gene expression data, as these are unable to distinguish trans-acting effects over local cis-acting effects when determining the actual causal genes. The candidate gene identified, *MTF2* (also known as *Pcl2*), is a metal response element binding transcription factor that selectively binds to unmethylated DNA [26] and has been found to control development of the anterior central nervous system [27]. Therefore, this demonstrates a previous role in brain development, making it a good candidate for further investigation as a regulator of inter-population variation in cerebrum size. The candidate genes identified for regulations of overall body mass were *PCBD2* and an EST (*603848039F1*). *PCBD2* is a target of the microRNA miR-3174, with this miRNA potentially having a role in rectal cancer through its action on *PCB2* [28]. The EST mapped to an exon on the gene *RCHY1* (ring finger and CHY zinc finger domain containing 1) that has been shown to have DNA damage response and cell cycle activity [29]. Additionally, evidence show that *HOXA2* induces *RCHY1* degradation [30], indicating that *RCHY1* might play a role in brain development as the HOX-genes are involved in embryonic development for both the axial skeleton and the hindbrain [31].

Although we did identify one candidate gene for cerebrum size (and two for body size), certainly many more were invariably failed to be identified. The fact that only one gene responsible for the variation in cerebrum size was identified could be due to the fact that the cerebrum is a large brain structure that consist of several substructures [13]. The functional constraints hypothesis (the mosaic hypothesis) predicts a more independent genetic basis for brain substructures than the concerted hypothesis and suggests that the correlated coevolution of brain regions sizes reflects the action of selection on functional systems connecting the different sub-components. The fact that we find very few correlations between the transcriptome of the cerebrum and the regulation of inter-population variation in size might therefore be due to the fact that the size of different sub-regions in the cerebrum is controlled by different genes and separate genetic architectures. Similarly, the effect size of these genes regulating this variation may also be too small to be detectable in our study. However, the link we do find between body-size and gene expression in the cerebrum suggest a link to the regulation of an individual’s overall size is partially regulated by the cerebrum. It was outside the scope of this study to do more detailed dissections on the cerebrum to measure size and gene expression in the smaller substructures, and our discussion on this matter therefore remains speculative.

To test the two different brain evolution hypotheses, a comparison between cerebrum and hypothalamus tissue was conducted. Relatively little overlap was identified between the two brain regions in terms of shared eQTL identified. In fact, both brain regions had more in common with the liver tissue, respectively, than each other. Similarly, when looking at shared eQTL hotspots, only two eQTL were present in hotspots in both types of brain tissue, with only one of those genes (*RHCY1* - EST *603848039F1*) actually having a similar genotypic effect (i.e. the Red junglefowl allele increasing expression) in both tissues. These results would seem to give greater evidence to the mosaic evolution hypothesis. In particular, the lack of shared eQTL between the two brain regions implies that genetic regulation of gene expression appears to be relatively independent between the two brain regions. Given the large differences in both overall brain size and composition between wild and domestic chickens, this large variation appears to share distinct regulatory patterns. However, there are several caveats to this interpretation. eQTL analysis, and indeed any gene expression analysis is of course dependent not only on the tissue but also on the time-point of sampling. As such it is possible that earlier sampling (for example immediately pre-hatch or early post-hatch) may have revealed a different genetic architecture of gene regulation, with more shared eQTL. In particular we know that brain composition changes from hatch to early adolescence to adulthood in chickens [5]. Similarly, an inherent problem with QTL mapping is that eQTL of small effect may well be missed, and as such some overlaps may be missed as a result.

The lack of shared eQTL hotspots between hypothalamus and cerebrum tissue also has some bearing on the Neural Crest Cell (NCC) hypothesis of domestication [32]. The NCC hypothesis conjectures that the migration and distribution of neural crest cells control a whole range of domestication-related traits, therefore pleiotropy is to be expected for multiple domestication-related QTL traits where they are all underpinned by loci controlling neural crest cell parameters. However, we find that rather few eQTL overlap between multiple tissue types (especially when we consider overlap between all three tissues). When we consider shared eQTL hotspots between the different brain tissues, there is some overlap, however only two actual eQTL are shared between the hotspots present in these two tissues. Interestingly, one of these genes, *GEMIN2* (also known as *SIP1*) has been associated to neural crest activity [33, 34] and motor neuron activity [35]. The other gene, *C5H15orf41,* has an unknown cellular function, though the open reading frame in *C5H15orf41* is highly conserved in vertebrates and has orthologs in human (C15orf41), rats (RGD1563680), mouse (BC052040), dog (C30H15orf41) and zebrafish (zgc:154061). Therefore, although the eQTL evidence overall does not appear to support neural crest cells as a mechanism of domestication, the presence of one gene does warrant further investigation in the future for a potential pleiotropic role.

## Conclusion

Overall our findings show greater support for the mosaic brain hypothesis (greater overlap in gene expression between liver and brain than between brain regions), however there are some results that could also indicate a role for the concerted brain evolution hypothesis (the identification of two candidate genes for body size detected in cerebrum tissue). Similarly, we also find less support for the neural crest cell hypothesis of domestication, with low replication of eQTL between multiple tissue types. Once again though, one shared eQTL was related to neural crest activity, and the identification of this gene’s exact role in domestication would be enlightening as regards the neural crest hypothesis. Finally, we also identify a candidate gene that is worthy of further investigation for regulating variation in cerebrum size. Given the paucity of genes responsible for regulating brain size variation that have been identified to date, the role of this gene is relevant to further our understanding of the molecular basis of avian brain evolution.

## Methods

### Study population and cross design

The chicken population used in this study was an eight-generation intercross started in the 1990s by crossing a Red Junglefowl rooster of Thai origin to three White Leghorn layer hens to generate 41 F_1_ individuals. The F_1_ were crossed to generate 811 F_2_ individuals, while F_3_ through F_7_ were kept at around 100 individuals. A total of 59 (26 females and 33 males) F_8_ individuals were used in this study, with the cerebrum (and hypothalamus [17]) dissected out at 212 days of age and snap frozen in liquid nitrogen and stored at minus 80 °C until further analysis. The F_8_-cross used in this study was reared at the research station of Linköping University, Sweden and maintained on standardized conditions and fed ad libitum, under a 12∶12 h light/dark regime. Housing pens measured 2.5 m × 2.5 m and where comprised of three separate levels equipped with perches [36, 37]. All individuals were culled by cervical neck dislocation followed by decapitation (as per the ethical permit). Animal handling was as per the ethical permit for the project. The study was approved by the local Ethical Committee of the Swedish National Board for Laboratory Animals, ethical permit Dnr 50–13.

### Phenotyping – brain measurements and dissections

Animals were weighed at hatch, 8 days, 42 days, 112 days, and 212 days. Immediately after culling at age 212 days, the brains were removed from the birds, and a four-piece dissection was performed. This involved dividing the brain into the cerebrum, optic tectum, cerebellum and a “midbrain” region (which included thalamus, the rest of the midbrain and the hindbrain; for more information on the dissected brain regions see [5]. After weighing each brain region separately to its nearest 0.001 g right after dissection, using a high precision balance (Sagitta 210 g/1 mg) the hypothalamus was dissected out of the “midbrain” regions.

### Genotyping

#### RNA isolation and gene expression microarrays

RNA was extracted from the cerebrum following a standard TRIzol-protocol with the quality assessed using a Bioanalyzer 2100 (Agilent). Samples with RIN values above 9 were used. RNA was converted to cDNA with Agilent one-color Low Input Quick Amp Labeling Kit using Cyanine 3-CTP. The labeled samples were hybridized on Agilent 8x60K custom gene expression microarrays following the manufacturer’s protocol and subsequently scanned on a NimbleGen MS200 (Roche NimbleGen) scanner. The microarray probesets were based on Ensembl transcripts and RefSeq mRNA sequences, with a total of 20,771 probesets representing 11,776 annotated genes. For further details about the microarray design see Johnsson et al. (2018) [18]. 2–3 probes were used for each probeset and summarised to one value per probeset using the R-package ‘preprocessCore’ [38]. Agilent Feature Extraction software v 12.0 was used to normalize and remove background noise from the microarray. To remove any slide batch effects, which can potentially occur when running multiple samples on the same slide, the R-package ‘sva’ was used [39]. A final clean up step was performed for the gene expression values where outliers were removed using the R-package ‘outliers’ with the chisq.out.test-function (*p*-value <1e-6, max 2 values were removed per probeset). All microarrays used in this study are available on ArrayExpress (http://www.ebi.ac.uk/arrayexpress) under the accession number E-MTAB-9313 (cerebrum), E-MTAB-3154 (hypothalamus) and E-MTAB-5572 (liver).

#### QTL and eQTL mapping

All chickens (*n* = 59) were genotyped. The DNA from each individual was prepared by Agowa (Berlin, Germany), using a standard salt DNA extraction [40]. A total of 612 informative SNP markers for genotyping were selected generating a 9667 cM genetic map and averaging 16 cM between each marker. Of these 551 markers were fully informative of parental origin, with the remainder being partially informative. Only genetic markers on the autosomes were used for QTL mapping. Note that this study presented here is a classical linkage study (using linkage between markers to map recombinations that occur between two intercrossed populations in a fixed series of inter-cross generations) [19], as opposed to a Genome Wide Association Mapping study (that uses the linkage disequilibrium that exists in a single natural population which has a built-up historical recombinations over a long period of time). The advantage of a linkage study means that the genome is covered using relatively few markers, and in fact very little is gained from having a density of markers less than 10 cM, whilst the recommended marker density for standard QTL mapping is 20-30 cM [41]. In the study presented here the average marker density is ~ 16 cM, meaning we have excellent genomic coverage to detect QTL with the number of SNPs that were used. The R-package ‘R/qtl’ was used for interval mapping with Haley-Knott regression [42]. Batch and sex were included in the models as fixed additive covariates and sex-interaction as interactive covariate. Any eQTL located near a target gene were considered as cis if the closest flanking markers was located at least 50 cM upstream and downstream of the gene. Determining whether a cis eQTL is acting on the target gene rather than an enhancer affecting the target gene is problematic and strictly should be called ‘local’ eQTL. However, this is a common problem in eQTL mapping [17] and any eQTL found within 100 cM of the target gene will hereafter be referred to as ‘cis’. Genome-wide trans eQTL were any eQTL outside the cis boundaries. Genes located on the Z chromosomes were included in the eQTL mapping. The power of the study (as calculated using the r/qtlDesign package [43]) was sufficient to give an 80% chance to detect a QTL of 27% effect size. Although these effects sizes are quite large, the nature of eQTL mapping means that the phenotypes used (gene expression) often have a higher r-squared in general in QTL mapping. Of course, these thresholds means that eQTL of small effect may well be missed. Phenotype and genotype data for the eQTL analysis is available as three data files in the supplementary information for this article (Supplementary Datasets: gene_expression_phenotypes.txt, genotypes.txt, and phenotypes.txt).

#### Significance thresholds for eQTL mapping

Logarithm of odds (LOD) thresholds were calculated using permutation tests [44], with 1000 permutations choosing 1000 probesets randomly over 10 iterations. The top 95th percentile was used as significant and the top 80th percentile as suggestive. Sex-interaction was taken into account. This generated a *trans* suggestive threshold of 4.55 and 6.52, nonsex and sex-interaction respectively, and significant threshold of 7.26 and 9.42, nonsex and sex-interaction respectively. The cis permutation was done on the immediate region of the SNP marker stretching to the closest flanking marker at least 50 cM upstream and downstream. This generated a cis suggestive threshold of 3.44 and 5.42, nonsex and sex-interaction respectively, and significant threshold of 3.75 and 5.96, nonsex and sex-interaction respectively. Confidence intervals (CI) for eQTL were calculated with a 1.8 LOD drop using the lodint*-*function (R/qtl) from the QTL peak to the closest marker and measured in genetic distance. This interval gives a 95% confidence interval in an intercross population [45].

### Analysis of candidate genes for cerebrum size

From a previous study phenotypic QTL for body and brain size were identified [5] and were overlapped with cerebrum eQTL identified in this study. These overlaps were tested on the confidence intervals between the eQTL and QTL. A linear regression was modelled between the overlapping trait as the response variable and gene expression as the predictor, with batch and sex as covariates. The *p*-values for the regression coefficient were Bonferroni corrected for the number of uncorrelated eQTL (gene expression values) within a trait QTL confidence interval.

### Network edge orientation analysis

To test for causality between gene expression and cerebrum size the software NEO was used [46]. The NEO software implements structural equation models (SEM) to fit multi-trait causal models in a trait network to find directionality between the traits. The genotype at each QTL peak was used as edges for the trait network and a causal model in which the genotype affects the trait using changing gene expression was compared to four alternative models, with these being the causal, reactive, confounded and collider models. For more information about the different models see [47, 48]. The best-fitting model is selected based on the ratio of the *χ*^2^*p*-value to the *p*-value of the next best-fitting model and referred to as local edge orienting against the next best model (leo.nb) scores. A positive leo.nb score indicates that the causal model fits better than any competing model. Aten et al. [46] use a single-marker leo.nb score of 1, corresponding to a 10-fold higher *p*-value of the causal model, and a multiple genetic marker leo.nb.oca of 0.3 as their threshold.

### Cerebrum gene profile compared to neuronal-and non-neuronal tissue

From previous studies done on the same intercross 1123 eQTL have been identified for the hypothalamus [17] and 1462 eQTL for liver [18]. These eQTL were used to compare gene expression profiles between the tissues (Cerebrum vs Hypothalamus, Cerebrum vs Liver, Hypothalamus vs. Liver and finally between all three tissues at once). Only eQTL found on the autosomes were used. As the studies were made on different custom expression arrays probesets found on both arrays were used in the analysis, in total 18,499. The resulting gene lists found to overlap between the tissues were tested for enrichment using the DAVID database v6.8 (https://david.ncifcrf.gov/) [49]. A custom background gene list was used composed of the genes present and expressed in the microarray (see supplementary Table S7) to avoid sampling bias [50]. Any functional aggregation of genes with a nominal *p*-values < 0.05 were reported, with genes that passed this threshold considered to be suggestive. Additionally, the more stringent Benjamini-Hockberg *p*-values < 0.05 (experiment-wide threshold) were also reported, with these considered significant.

### eQTL hotspots

Regions on the genome were sought after where the observed number of eQTL was higher than expected. These regions, termed hotspots, indicate transcriptionally active regions specific for our study population. eQTL hotspots were assigned by overlapping the eQTL confidence intervals throughout each autosomal chromosome. Chromosomes with at least 10 eQTL were considered. A permutation test was used to set the significance threshold, this was done for each chromosome individually. The average confidence interval for the eQTL was randomly assigned a position on the chromosome equal to the number of eQTL found on the chromosome. The highest number of overlaps was saved. This was repeated 1000 times and the 95th percentile used as threshold. Additionally, the eQTL hotspots and their corresponding genes were tested for enrichment using the DAVID database v6.8 (https://david.ncifcrf.gov/) [49], using the same custom background gene list as above.

## Supplementary information

**Additional file 1: Table S1.** All eQTL present in the cerebrum tissue. For each eQTL, the chromosome, position (in cM), LOD score, confidence interval, direction of effect (whether the allele with the greater effect derived from the RJF or WL genotype), % of variance explained by the eQTL, the additive and dominance effects of each eQTL, plus the additive and dominance effects if a sex-interaction was also present, as well as information on the gene underlying each eQTL (whether it represented an annotated gene and the bp position of the gene).

**Additional file 2: Table S2.** GO analysis for each of the eQTL hotspots. GO categories and terms are presented, along with the counts for each term, the nominal *p*-value (with this considered suggestive if *P* < 0.05), and the Benjamini-corrected *p*-value (with this considered significant if P < 0.05).

**Additional file 3: Table S3.** Overlaps between eQTL and phenotypic QTL. For each overlap, the phenotypic trait QTL, the eQTL probeset, the confidence intervals for the two QTL, and the eQTL type (whether it was a cis or trans eQTL) are shown.

**Additional file 4: Table S4.** Significant and suggestive overlaps between phenotypic QTL and eQTL. For each overlap, as well as the trait type and eQTL probeset, the % of variation in the phenotype explained by the probeset (adjusted R-squared), the correlation *p*-value (*p*-value of probeset expression in the model) and t-value, as well as the gene ID (ensgalg ID) are provided.

**Additional file 5: Table S5.** GO analysis for each of the tissue comparisons (pairwise overlaps between eQTL shared between cerebrum and liver, cerebrum and hypothalamus, hypothalamus and liver, and all three tissues. GO categories and terms are presented, along with the counts for each term, the nominal *p*-value (with this considered suggestive if *P* < 0.05), and the Benjamini-corrected *p*-value (with this considered significant if P < 0.05).

**Additional file 6: Table S6.** Shared genes possessing an eQTL expressed in multiple tissues. For each gene, the tissue comparison (i.e if the gene is shared between cerebrum and hypothalamus, cerebrum and liver, liver and hypothalamus, or all three tissues), Gal4 and Gal6 gene location, Gal4 and Gal6 annotation number and the probeset itself are given.

**Additional file 7: Table S7.** Gene lists used to generate the DAVID GO results.

**Additional file 8.** Gene_expression_phenotypes.

**Additional file 9.** Genotypes.

**Additional file 10.** Phenotypes.

## Data Availability

The microarray datasets generated during the current study are available in the ArrayExpress repository (http://www.ebi.ac.uk/arrayexpress) under the accession number E-MTAB-9313 (cerebrum), E-MTAB-3154 (hypothalamus) and E-MTAB-5572 (liver). Phenotype and genotype data for the eQTL analysis is available as three data files in the supplementary information for this article (gene_expression_phenotypes.txt, genotypes.txt, and phenotypes.txt).

## Abbreviations

EST: Expression sequence tags
DNA: Deoxyribonucleic acid
GWAS: Genome-wide association study
miRNA: microRNA
RJF: Red junglefowl
RNA: Ribonucleic acid
QTL: Quantitative trait locus
eQTL: Expression quantitative trait locus
WL: White Leghorn
NEO: Network Edge Orientation

## References

[1] Swanson LW (2000). Cerebral hemisphere regulation of motivated behavior. Brain Res.

[2] Güntürkün O, Bugnyar T (2016). Cognition without cortex. Trends Cogn Sci [Internet].

[3] Shimizu T, Shinozuka K, Uysal AK, Leilani KS (2017). The origins of the bird brain: multiple pulses of cerebral expansion in evolution. Evolution of the brain, cognition, and emotion in vertebrates [Internet].

[4] Rehkämper G, Frahm HD, Cnotka J (2008). Mosaic evolution and adaptive brain component alteration under domestication seen on the background of evolutionary theory. Brain Behav Evol.

[5] Henriksen R, Johnsson M, Andersson L, Jensen P, Wright D (2016). The domesticated brain: Genetics of brain mass and brain structure in an avian species. Sci Rep [Internet].

[6] Iwaniuk AN, Hurd PL (2005). The evolution of cerebrotypes in birds. Brain Behav Evol.

[7] Henriksen R, Wright D, Vonk J, Shackelford T (2017). Cerebrotype. Encyclopedia of animal cognition and behavior.

[8] Burish MJ, Kueh HY, Wang SSH (2004). Brain architecture and social complexity in modern and ancient birds. Brain Behav Evol.

[9] Montgomery SH, Mundy NI, Barton RA. Brain evolution and development: Adaptation, allometry and constraint. Proc R Soc B Biol Sci. 2016;283(1838):20160433. 10.1098/rspb.2016.0433 PMC5031648 27629025

[10] Hoops D, Vidal-García M, Ullmann JFP, Janke AL, Stait-Gardner T, Duchêne DA (2017). Evidence for concerted and mosaic brain evolution in dragon lizards. Brain Behav Evol.

[11] Moore JM, Devoogd TJ. Concerted and mosaic evolution of functional modules in songbird brains. Proc R Soc B Biol Sci. 2017;284(1854):20170469. 10.1098/rspb.2017.0469 PMC5443954 28490627

[12] Gutiérrez-Ibáñez C, Iwaniuk AN, Moore BA, Fernández-Juricic E, Corfield JR, Krilow JM, Kolominsky J, Wylie DR. Mosaic and concerted evolution in the visual system of birds. PLoS One. 2014;9(3):e90102. 10.1371/journal.pone.0090102 PMC3951201 24621573

[13] Jarvis ED, Yu J, Rivas MV, Horita H, Feenders G, Whitney O (2013). Global view of the functional molecular organization of the avian cerebrum: mirror images and functional columns. J Comp Neurol.

[14] Bélteky J, Agnvall B, Johnsson M, Wright D, Jensen P. "Domestication and tameness: Brain gene expression in red junglefowl selected for less fear of humans suggests effects on reproduction and immunology." R Soc Open Sci. 2016;3(8):160033. 10.1098/rsos.160033 PMC5108935 27853585

[15] Gering E, Incorvaia D, Henriksen R, Conner J, Getty T, Wright D (2019). Getting Back to Nature: Feralization in animals and plants. Trends Ecol Evol.

[16] Johnsson M, Gering E, Willis P, Lopez S, Van Dorp L, Hellenthal G (2016). Feralisation targets different genomic loci to domestication in the chicken. Nat Commun [Internet].

[17] Johnsson M, Williams MJ, Jensen P, Wright D (2016). Genetical genomics of behavior: a novel chicken genomic model for anxiety behavior. Genetics..

[18] Johnsson M, Henriksen R, Höglund A, Fogelholm J, Jensen P, Wright D (2018). Genetical genomics of growth in a chicken model. BMC Genomics.

[19] Lynch M, Walsh B (1998). Genetics and analysis of quantitative traits. Sunderland, Mass. Sinauer.

[20] Ikram MA, Fornage M, Smith AV, Seshadri S, Schmidt R, Debette S (2012). Common variants at 6q22 and 17q21 are associated with intracranial volume. Nat Genet.

[21] Stein JL, Medland SE, Vasquez AA, Hibar DP, Senstad RE, Winkler AM (2012). Identification of common variants associated with human hippocampal and intracranial volumes. Nat Genet.

[22] Hibar DP, Stein JL, Renteria ME, Arias-Vasquez A, Desrivières S, Jahanshad N (2015). Common genetic variants influence human subcortical brain structures. Nature.

[23] Hager R, Lu L, Rosen GD, Williams RW (2012). Genetic architecture supports mosaic brain evolution and independent brain-body size regulation. Nat Commun.

[24] Paus T, Bernard M, Chakravarty MM, Davey Smith G, Gillis J, Lourdusamy A (2012). KCTD8 gene and brain growth in adverse intrauterine environment: a genome-wide association study. Cereb Cortex.

[25] Adams HHH, Hibar DP, Chouraki V, Stein JL, Nyquist PA, Rentería ME (2016). Novel genetic loci underlying human intracranial volume identified through genome-wide association. Nat Neurosci.

[26] Perino M, Van Mierlo G, Karemaker ID, Van Genesen S, Vermeulen M, Marks H (2018). MTF2 recruits Polycomb repressive complex 2 by helical-shape-selective DNA binding. Nat Genet.

[27] Funck-Brentano C, Lancar R, Le Heuzey JY, Lardoux H, Soubrie C, Lechat P (2001). Xenopus Polycomblike 2 (XPcl2) controls anterior to posterior patterning of the neural tissue. Dev Genes Evol.

[28] Zhang D-L, Yang N (2019). MiR-3174 functions as an oncogene in rectal cancer by targeting PCBD2. Eur Rev Med Pharmacol Sci.

[29] Halaby MJ, Hakem R, Hakem A (2013). Pirh2: an E3 ligase with central roles in the regulation of cell cycle, DNA damage response, and differentiation. Cell Cycle.

[30] Bridoux L, Deneyer N, Bergiers I, Rezsohazy R (2015). Molecular analysis of the HOXA2-dependent degradation of RCHY1. PLoS One.

[31] Alexander T, Nolte C, Krumlauf R (2009). Hox genes and segmentation of the hindbrain and axial skeleton. Annu Rev Cell Dev Biol.

[32] Wilkins AS, Wrangham RW, Tecumseh FW (2014). The “domestication syndrome” in mammals: a unified explanation based on neural crest cell behavior and genetics. Genetics.

[33] Rogers CD, Saxena A, Bronner ME (2013). Sip1 mediates an E-cadherin-to-N-cadherin switch during cranial neural crest EMT. J Cell Biol.

[34] Yasumi T, Inoue M, Maruhashi M, Kamachi Y, Higashi Y, Kondoh H (2016). Regulation of trunk neural crest delamination by δEF1 and Sip1 in the chicken embryo. Develop Growth Differ.

[35] Zhang H, Xing L, Rossoll W, Wichterle H, Singer RH, Bassell GJ (2006). Multiprotein complexes of the survival of motor neuron protein SMN with Gemins traffic to neuronal processes and growth cones of motor neurons. J Neurosci.

[36] Johnsson M, Gustafson I, Rubin CJ, Sahlqvist AS, Jonsson KB, Kerje S, et al. A Sexual Ornament in Chickens Is Affected by Pleiotropic Alleles at HAO1 and BMP2, Selected during Domestication. PLoS Genet. 2012;8(8). 10.1371/journal.pgen.1002914 PMC3431302 22956912

[37] Johnsson M, Rubin CJ, Höglund A, Sahlqvist AS, Jonsson KB, Kerje S (2014). The role of pleiotropy and linkage in genes affecting a sexual ornament and bone allocation in the chicken. Mol Ecol.

[38] Bolstad BM, Irizarry RA, Åstrand M, Speed TP (2003). A comparison of normalization methods for high density oligonucleotide array data based on variance and bias. Bioinformatics..

[39] Johnson WE, Li C, Rabinovic A (2007). Adjusting batch effects in microarray expression data using empirical Bayes methods. Biostatistics..

[40] Aljanabi S (1997). Universal and rapid salt-extraction of high quality genomic DNA for PCR- based techniques. Nucleic Acids Res.

[41] Darvasi A, Soller M (1994). Optimum spacing of genetic markers for determining linkage between marker loci and quantitative trait loci. Theor Appl Genet.

[42] Broman KW, Wu H, Sen Ś, Churchill GA (2003). R/qtl: QTL mapping in experimental crosses. Bioinformatics.

[43] Broman KW, Sen S (2009). A guide to QTL mapping with R/qtl [Internet].

[44] Doerge RW, Churchill GA (1996). Permutation tests for multiple loci affecting a quantitative character. Genetics.

[45] Manichaikul A, Dupuis J, Sen Ś, Broman KW (2006). Poor performance of bootstrap confidence intervals for the location of a quantitative trait locus. Genetics..

[46] Aten JE, Fuller TF, Lusis AJ, Horvath S (2008). Using genetic markers to orient the edges in quantitative trait networks: the NEO software. BMC Syst Biol.

[47] Fogelholm J, Inkabi S, Höglund A, Abbey-Lee R, Johnsson M, Jensen P (2019). Genetical genomics of tonic immobility in the chicken. Genes (Basel).

[48] Johnsson M, Henriksen R, Fogelholm J, Höglund A, Jensen P, Wright D (2018). Genetics and genomics of social behavior in a chicken model. Genetics.

[49] Huang DW, Sherman BT, Lempicki RA (2009). Systematic and integrative analysis of large gene lists using DAVID bioinformatics resources. Nat Protoc.

[50] Timmons JA, Szkop KJ, Gallagher IJ (2015). Multiple sources of bias confound functional enrichment analysis of global -omics data. Genome Biol.

